# Analysis of Pollution in High Voltage Insulators via Laser-Induced Breakdown Spectroscopy

**DOI:** 10.3390/molecules25040822

**Published:** 2020-02-13

**Authors:** Xinwei Wang, Shan Lu, Tianzheng Wang, Xinran Qin, Xilin Wang, Zhidong Jia

**Affiliations:** 1 Shanxi Electric Power Research Institute, Taiyuan 030000, China; wxw7912@163.com (X.W.); ls8760033@163.com (S.L.); wtz2000@163.com (T.W.); 2 Engineering Laboratory of Power Equipment Reliability in Complicated Coastal Environments, Tsinghua Shenzhen International Graduate School, Shenzhen 518055, China; txr19@mails.tsinghua.edu.cn (X.Q.); jiazd@sz.tsinghua.edu.cn (Z.J.)

**Keywords:** laser-induced breakdown spectroscopy, surface pollution, high voltage insulators, quantitatively analysis

## Abstract

Surface pollution deposition in a high voltage surface can reduce the surface flashover voltage, which is considered to be a serious accident in the transmission of electric power for the high conductivity of pollution in wet weather, such as rain or fog. Accordingly, a rapid and accurate online pollution detection method is of great importance for monitoring the safe status of transmission lines. Usually, to detect the equivalent salt deposit density (ESDD) and non-soluble deposit density (NSDD), the pollution should be collected when power cut off and bring back to lab, time-consuming, low accuracy and unable to meet the online detection. Laser-induced breakdown spectroscopy (LIBS) shows the highest potential for achieving online pollution detection, but its application in high voltage electrical engineering has only just begun to be examined. In this study, a LIBS method for quantitatively detecting the compositions of pollutions on the insulators was investigated, and the spectral characteristics of a natural pollution sample were examined. The energy spectra and LIBS analysis results were compared. LIBS was shown to detect pollution elements that were not detected by conventional energy spectroscopy and had an improved capacity to determine pollution composition. Furthermore, the effects of parameters, such as laser energy intensity and delay time, were investigated for artificial pollutions. Increasing the laser energy intensity and selecting a suitable delay time could enhance the precision and relative spectral intensities of the elements. Additionally, reducing the particle size and increasing the density achieved the same results.

## 1. Introduction

The insulators were key equipment in transmission lines, in order to mechanically support conductor and give enough insulation space between conductor and tower. After being in operation for in a transmission line, an insulator (ceramic, glass or composite insulator) can accumulate a thick layer of pollutants on its surface due to different environmental factors. Under dry conditions, pollution was not harmful and had little effect on the safe service. However, soluble pollutants can be dissolved in water, forming a conductive water film on the surface of an insulator; this process results in the formation of conductive channels on the surface of the insulator, and in turn, reduces the pollution flashover voltage (PFV), thereby causing partial discharge, arc and even flash-over incidents [1]. Methods for detecting the pollution characteristics and pollution level of insulators have been studied for a long time. The Working Group 04 of Study Committee 33 (Over-voltage and Insulation Coordination) of the International Council on Large Electric Systems has recommended five methods for quantitatively characterizing pollution levels, including the equivalent salt deposit density (ESDD), surface conductivity, leakage current, PFV and pollution flashover gradient.

Pollution composition is complex and differs between environments. In nature, soluble pollutions are primarily conductive electrolytes, such as NaCl, KCl, CaSO_4_, CaCl_2_, Na_2_SO_4_, NaNO_3_ and KNO_3_; the main insoluble pollutions include SiO_2_, C, Al_2_O_3_, MgSO_4_, Fe_2_O_3_ and CaO [2,3]. Researchers have found that the pollution levels measured by ESDD differ from the actual values to a certain extent. As a result, the PFVs of artificial pollutions are lower than those of natural pollutions with the same ESDD. The PFV of the artificial pollution CaSO_4_ is higher than that of the artificial pollution NaCl for the same ESDD. Additionally, for an artificial pollution mixture of NaCl and CaSO_4_, the higher the CaSO_4_ content, the higher the PFV is [4,5,6,7].

Currently, researchers also employ other indirect methods (e.g., light, sound and electricity) to determine the pollution levels. Hyperspectral imaging, microwave radiation theory, infrared and visible light information fusion, ultraviolet sensors, light detection sensors and acoustic emission technology have been employed to establish insulator pollution level prediction models [8,9,10,11,12,13,14,15]. With respect to the direct detection of pollution composition, aside from commonly used material composition analysis methods (e.g., ion emission spectroscopy techniques, including ion chromatography, X-ray diffraction and inductive coupling), very few researchers have examined online detection methods for insulator pollution composition. However, the compositional distribution of pollutions on surface of an insulator is often complex and heterogeneous. These factors present difficulties for evaluating pollution levels by indirect methods. Additionally, research results have demonstrated that pollution composition and material characteristics can affect the pollution flashover process, and may cause excessive or deficient insulation in insulation design [16,17,18]. The PFV is not only related to soluble salt composition, but also affected by insoluble substances in different mixtures [19].

To improve the accuracy and application of LIBS. The researchers studied various sample preparation techniques, such as dilution and using binding material, etc. By milling [20] and grinding, the particle size is reduced and the surface area is increased to make the sample more uniform. The smaller the particle size, the easier it is to evaporate and atomize in the plasma [21].

Laser-induced breakdown spectroscopy (LIBS) is a qualitative and quantitative analytical method based on pulse laser technology that examines the plasma atomic emission spectrum after exciting the sample [22], and it had higher sensitivity for light elements detection (H,Li,C,Si etc.), compared to EDS (or EDX) technique [23,24]. Currently, owing to the rapid development of this technique, the use of LIBS is widespread in the theoretical and experimental research of many fields, such as those of mineral products, archaeology, biomedicine and aerospace exploration [25,26,27,28,29]. In particular, LIBS is currently the only feasible technique in fields that require remote elemental analysis [26]. We have [30,31] evaluated the feasibility of using LIBS to achieve rapid, accurate, online monitoring of the ageing performance of silicone rubber and to determine the components (C, O, Fe and Si) that are closely related to the ageing state of silicone rubber. Combined with XPS technology, the linear calibration curves of these components were established. Based on the variation trend of element spectral intensity with depth, the depth of aging layer was obtained. However, compared with silicone rubber, pollution composition is more complex and varied. Therefore, when studying pollutions using LIBS, it is necessary to consider the effects of the properties of the pollutions and optimize the system parameters. In this study, the effects of various factors on the LIBS spectra of natural and artificial pollutions are examined and optimized system parameters are proposed.

## 2. Results and Discussion

### 2.1. Microanalysis and LIBS Testing of Pollutions Sampled

**Figure 1:**
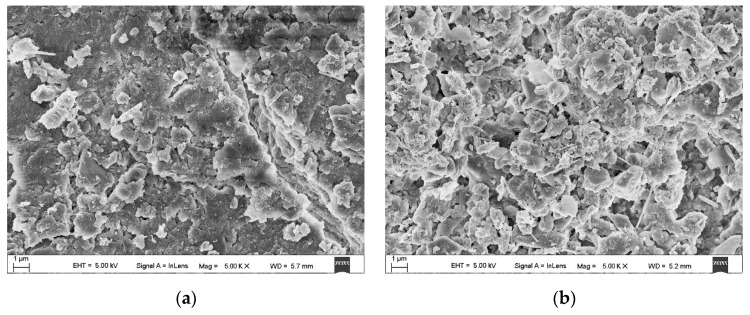
Scanning electron microscopy (SEM) images (5000×) of the natural pollutions on the surface of the insulator (**a**) Sampling point 1, (**b**) sampling point 2.

Figure 1 shows SEM images of the natural pollutions on the surface of the insulator at two randomly selected analytical points. As demonstrated in Figure 1, the pollutions at sampling point 1 were densely distributed and exhibited layer-by-layer stacking. In contrast, the pollutions at sampling point 2 were loosely arranged, and there were relatively large spaces between the pollutions. The process by which natural pollutions were adhered to the surface of an insulator is affected by the air flow in the environment. The uneven adhesive forces between particles and the surface of an insulator, due to the ageing of the insulator and the random interactions between particles, can result in uneven adherence of pollutions on the surfaces of adjacent insulators.

The EDS detector of the SEM was used to analyze the elements on the surface of the insulator. Table 1 summarizes the results. Very few elements were detected by EDS, and minimal Cl was detected. Natural pollutions often contain NaCl and KCl, which significantly affect pollution flashovers. The NaCl and KCl on the surface of the insulator may have been eliminated by dissolution and scouring as a result of dampening and rainfall. The EDS detector only analyzed the surface composition of the sample and consequently failed to detect the distributions of other common elements. Titanium ore is in the area of insulator operation, so there is high concentration of Ti in the pollution. Therefore, other methods were needed to determine the composition of pollutions on the surface of the insulator.

**Figure 2:**
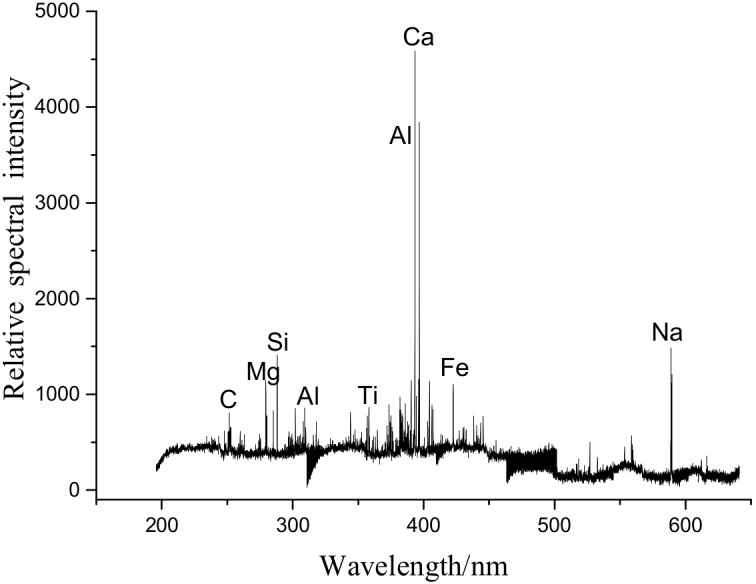
LIBS spectrum of the natural pollutions.

Figure 2 showed the LIBS spectrum of the natural pollutions. Table 2 showed the wavelength of typical spectral lines in Figure 2. In Figure 2, the wavelengths of the abscissa correspond to the emission intensities of various elements. Each element has multiple emission lines. In testing, a characteristic wavelength should be selected, and the type of element and relative spectral intensity, corresponding to the characteristic wavelength, should be determined [32]. Spectral intensities reflect the composition of the sample tested. As shown in Figure 2, Si, Ca, Al, C and Na had relatively high intensity, and this indicates that, agreeing well with the EDS area scan results, the natural pollutants had relatively high contents of these elements.

Trace amounts of Na and Mg were detected in the samples tested by LIBS, while the EDS area scans of the sample, Na and Mg were not detected in the ablation pits of the silicone rubber. Therefore, LIBS can not only achieve rapid, online detection of elements, but also help further reduce the detection limit of current composition testing and improve the accuracy of quantitative/qualitative compositional analysis.

### 2.2. Effects of Single-Pulse Laser Energy on the LIBS Signal

**Figure 3:**
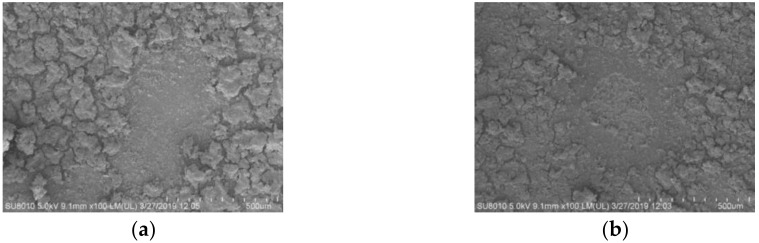
Distribution of the natural pollutions on the surface of the insulator (**a**) SEM image of the entire ablated sample top, (**b**) SEM image of the entire ablated sample bottom.

In LIBS, the depth of ablation craters depends on many factors, such as laser energy, ablation duration and material characteristics. The single-pulse laser energy has an impact on the ablation of pollutions on the surfaces of insulators. Ideally, a single-pulse laser beam only ablates the pollutions on the surface of an insulator but not the surface of the insulator itself. Figure 3 shows SEM images (200×) of the laser-ablated samples (labelled top and bottom). As demonstrated in Figure 3a,b, a focused laser beam produced an ellipsoidal ablation pit on the surface of each sample, which was related to the morphology of the focused laser beam. The sample bottom was taken from the surface of a silicone rubber insulator that had aged as a result of being in service for an extended period of time, and cracks differing in size were distributed on its surface. To further analyze the ablation effects of a single-pulse laser on the natural pollutions on the surface of the insulator, EDS area scans were performed on the natural pollutions and the ablation pits on the surfaces of the samples top and bottom to analyze the elemental compositions. The results showed that the typical characteristic elements (e.g., Na, Mg and Ti) were not detected in these samples by EDS. This observation suggests that the LIBS testing, with a single- pulse laser beam with an output energy of 110 mJ, was able to penetrate the relatively thin (micro-sized) pollution layers and ablated the pollutions at the point of action into laser plasma, thereby, exposing the substrate of the insulator.

**Figure 4:**
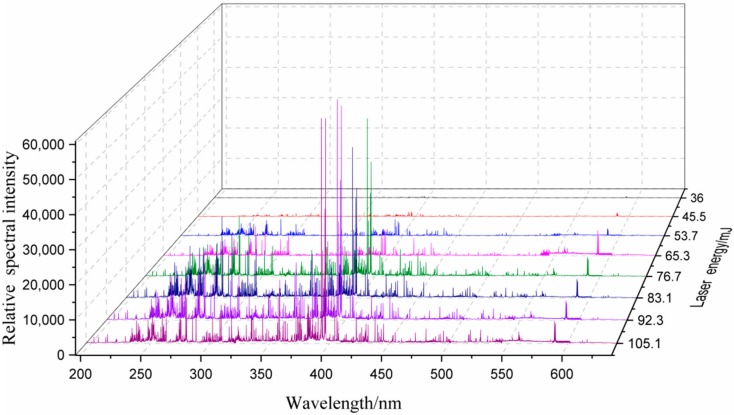
LIBS spectra within a certain band range under various laser energies.

A laser energy increases within a certain range, the energy absorbed per unit target surface area increases, resulting in an increase in the spectral intensity of the sample. Once the increase in laser energy outside this range may result in self-absorption of or matrix effects on elements, which in turn, results in a decrease in intensity. In the experiment, artificial pollutions were prepared to determine the spectral intensities under various laser energies within a reasonable range. Figure 4. shows partial LIB spectra, obtained under various strengths of laser energies. As demonstrated in Figure 4, as the laser energy increased, the spectral intensities corresponding to different wavelengths increased by varying degrees. The spectral intensity of Al corresponding to a wavelength of 396.592 nm saturated prematurely. Therefore, while higher laser energy may improve the spectral intensity, extremely high laser energy outranging a certain range may interfere with the experiment.

**Figure 5:**
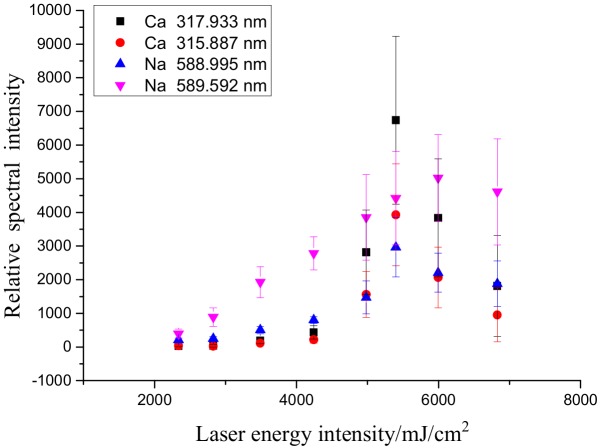
Effects of laser energy on the spectral intensities of the elements tested

**Figure 6:**
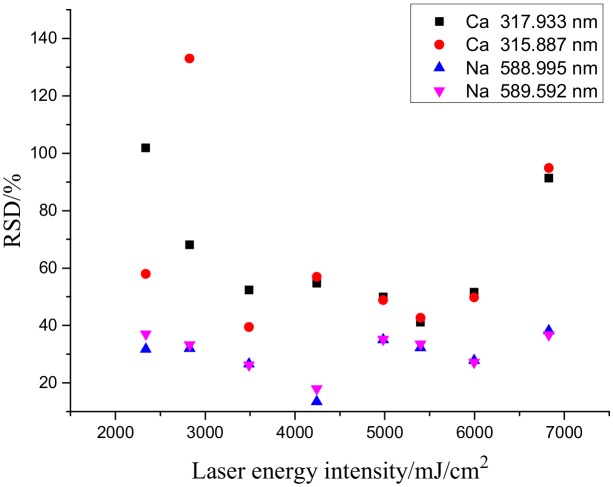
Relationship between the relative spectral intensity and measurement repeatability of the elements tested.

This work done with the insulator that has been exposed to the elements. The LIBS method was described as follows: Five points on the surface of each sample were randomly selected. Each point was subjected to five continuous laser treatments. Figure 5 and Figure 6 show the effects of laser energy density on the spectral intensity, and relative standard deviation (RSD) of various elements tested, respectively. The laser energy intensity was obtained by dividing the laser energy by the spot area. The diameter of laser focusing on sample surface was 0.8 mm. The spectral line intensity increased as the pulsed laser energy intensity increased. As demonstrated in Figure 6, as the spectral intensity increased, the RSDs of almost all the elements gradually decreased, suggesting that increasing laser energy intensity could effectively improve the repeatability of results. The RSD is related to the concentration and spectral line intensities of the sample and is affected by the spectral analysis conditions and instrument performance.

Additionally, an increase in laser energy intensity provided sufficient excitation energy for certain elements, causing intensity saturation or self-absorption effects and consequently decreasing the peak values. Meanwhile, owing to matrix effects, the increase in laser energy significantly interfered with the spectral information of other elements, leading to negative effects. Based on the SEM results for the pollutions subjected to LIBS testing, the laser energy was adjusted to approximately 80 mJ, corresponding to a laser ablation density of 3.814 × 10^10^ Watts/cm2. The ablation effects of the adjusted laser energy on the surface of the composite insulator were comparatively analyzed.

### 2.3. Selection of Delay Time

**Figure 7:**
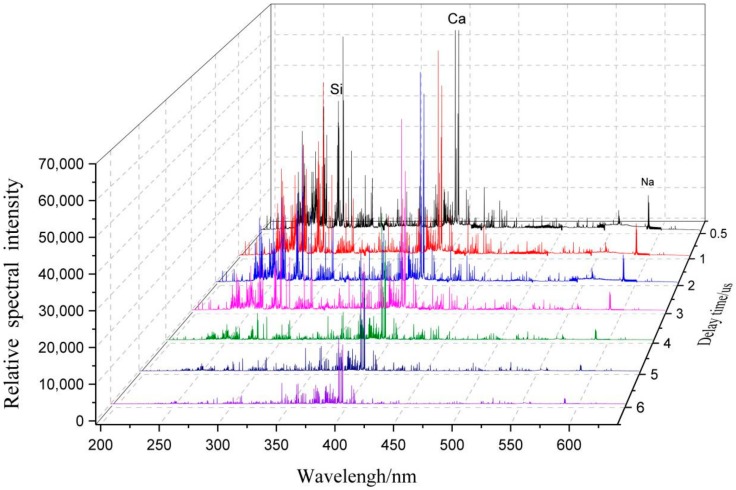
Changes in LIBS spectra within a certain band range with delay time.

**Figure 8:**
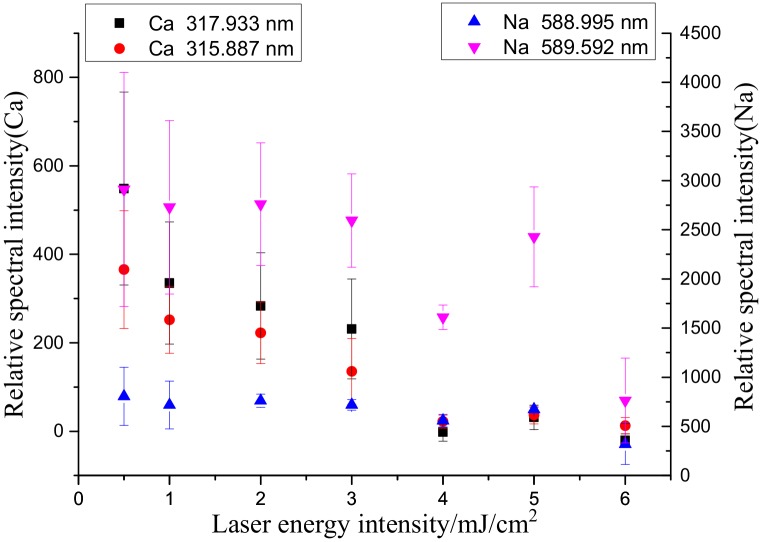
Effects of delay time on the relative spectral intensity of each element.

Figure 7 shows the trends of the spectral intensities within the same band range with the delay time. As demonstrated in Figure 7, as the delay time increased, the normalized spectral intensity corresponding to each wavelength significantly decreased. Using the average relative spectral intensity at a delay time of 0.5 µs as the baseline, the normalized relative spectral intensities were calculated by dividing the average relative spectral intensities at other delay times by the baseline. Figure 8 shows the results, the experimental results show that the trends of the spectral intensities of each element corresponding to various wavelengths were similar. Hence, only the spectral intensity of one element, corresponding to one wavelength, was selected for analysis.

**Figure 9:**
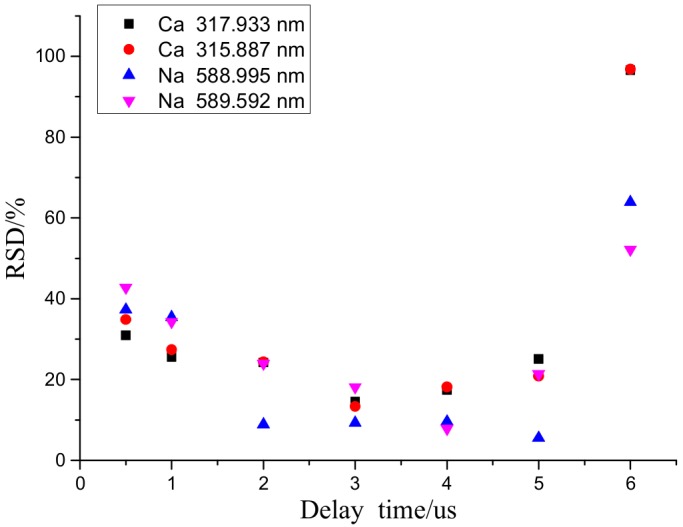
Effects of delay time on the repeatability of measurements on the elements tested.

As demonstrated in Figure 8, the continuous background spectral process was not complete at a delay time of 0.5 μs. As the delay time increased, the spectral intensity of each element considerably decreased. Additionally, as the delay time increased, the RSD for Ca first slowly increased, and then gradually stabilized as shown in Figure 9. During the plasma cooling process, the collisions between ions and electrons continuously weakened, and consequently, the luminous intensities of energy released from the collisions and received by the spectrometer continuously decreased. In particular, as the delay time increased from 1 to 9 µs, the normalization ratio for Na fluctuated in the range of 0.3–0.45 because Na, being an alkali metal element prone to ionization, was completely ionized within 1 µs. As a result, as the measurement delay time increased, the number of Na ions received by the system decreased, resulting in a decrease in the measurement accuracy.

Considering the relationships among the spectral intensity, RSD and delay time, a delay time ranging from 2 to 4 µs was selected as the optimum delay time range that led to a normalization ratio greater than 0.4 and an RSD less than 20%. A gate-width delay time of 3 µs was used in the subsequent experiment. When analyzing a particular element, a delay time range that leads to a normalization ratio greater than 0.5 and a minimum RSD should be selected.

### 2.4. Effects of Pollution Particle size and Density on LIBS Signal

Pollutions on the surface of an insulator in operation have complex and varied compositions (as shown in Figure 1). Pollution particles vary in size between different locations, and the gap density varies between pollutions. Inconsistent particle sizes and densities can both affect LIBS spectra.

First, the effects of pollution particle size on the LIBS spectral signal were studied. A Malvern Mastersizer 2000 laser particle-size analyzer was used to measure the particle size of the NaCl samples and kaolin clay [33]. A wet method was employed, and ethanol was used to dissolve the samples. Table 3 summarized the particle-size test results.

**Figure 10:**
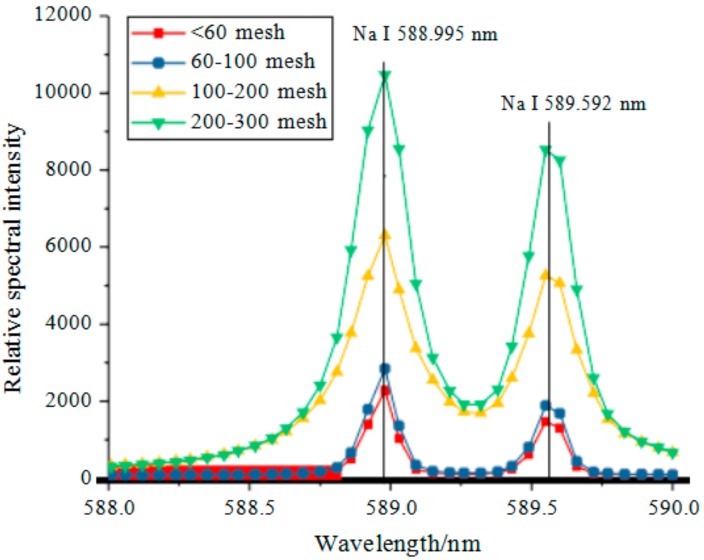
Spectral intensities of Na at 589 nm.

The LIBS method was described as follows: Five points on the surface of each sample were randomly selected. Each point was subjected to five continuous laser treatments (frequency: 1 Hz). The relative spectral intensities of Na corresponding to wavelengths of 588.995 and 589.592 nm were extracted from the LIBS spectrum acquired for data analysis. The spectral intensities from 25 points on each sample were each divided by the background signal of the substrate, and the results were averaged (shown in Figure 10). As demonstrated in Figure 10, as the NaCl particle size decreased, the spectral intensities corresponding to the wavelengths of 588.995 and 589.592 nm gradually increased.

**Figure 11:**
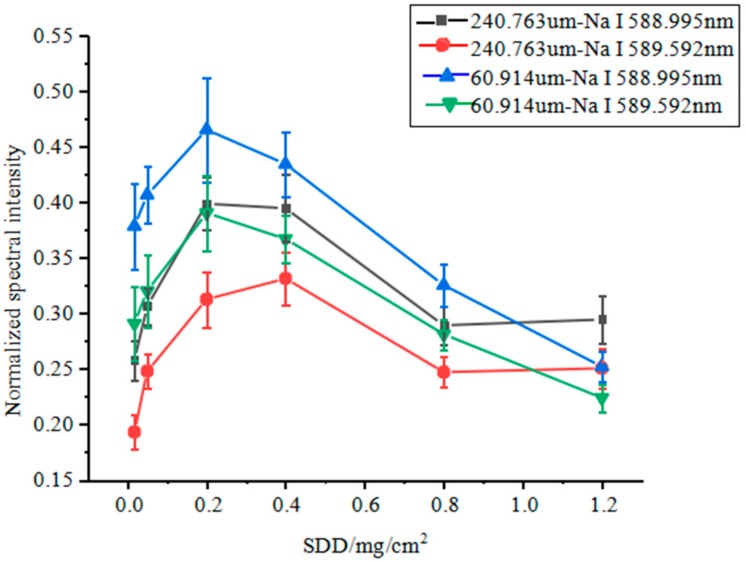
Changes in the spectral intensities of Na in NaCl samples differing in particle size with NaCl concentration.

Based on the total area of XP-70, the mass of NaCl in each sample was determined. Each sample was compressed using a compression machine and subsequently subjected to LIBS testing. Figure 11 shows the changes in the relative spectral intensities of the Na in NaCl samples differing in particle size with NaCl concentration. When the NaCl particle size remained constant, the average relative spectral intensity of the two spectral lines of Na first increased and then decreased, as ESDD increased. For the NaCl samples with the same ESDD, the spectral intensity of Na was higher in the NaCl sample with a particle size of 60.914 μm than that in the NaCl sample with a particle size of 240.764 μm, exhibiting a trend similar to that in Figure 8.

**Figure 12:**
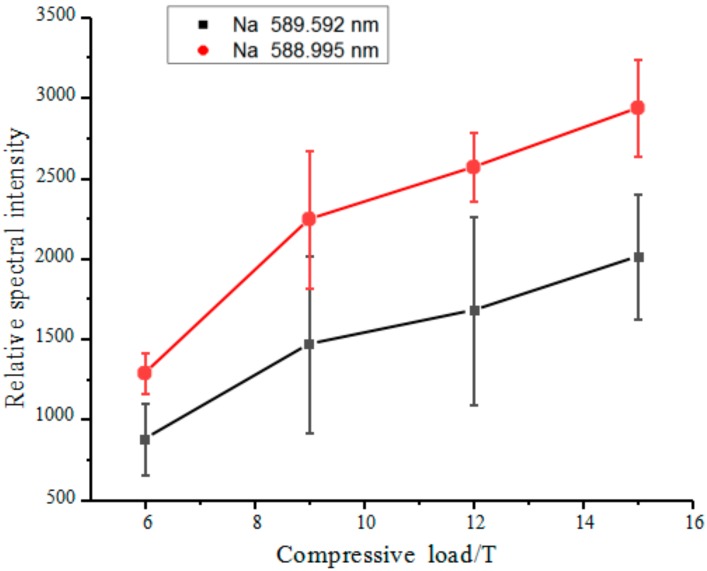
Effects of density on spectral intensity.

Density is one of the variable properties of pollutions. Pollutions on the surfaces of insulators in different operating environments vary significantly in density. Thus, it is necessary to examine the effects of pollution density on LIBS spectral signals. Four identical artificial pollution samples, each consisting of kaolin clay (2 g) and NaCl (1%), were prepared. The four samples were compressed using a compression machine under compressive loads of 6, 9, 12 and 15 t. The compressed samples were subsequently subjected to LIBS testing to determine the relationship between compressive load and average relative spectral intensity (Figure 12). As demonstrated in Figure 12, as the density increased, the relative excited spectral intensities of the samples increased. This phenomenon can be explained by excited plasma plume dynamics. When the laser energy acts on the surface of a sample, the denser the surface of the sample is, the greater the impact of the laser pulse reverse shock wave is. Various types of particles jet from the target surface opposite the direction of the laser. The increase in the reverse jet velocity and intensity of various types of particles strengthens the collision ionization during the rapid expansion of the plasma, thereby, improving the atomic emission intensity.

## 3. Experiments

**Figure 13:**
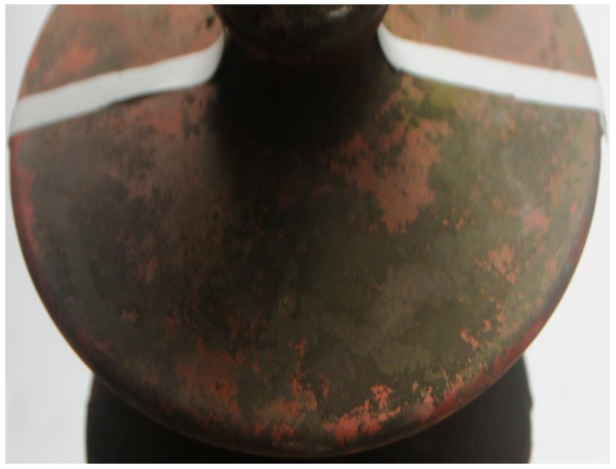
Schematic diagram of the insulators used in the experiment.

A composite insulator chain (manufactured by Dongguan Gaoneng Industry Co., Ltd. in Dongguan, China) was collected from the N63 jumper of the 220-kV Dongguan–Kuihu line A. As shown in Figure 13, an insulator was cut from the centre of the insulator chain along the external surface of the core of the chain. A small piece (1 cm × 1 cm) was cut from a relatively dark-colored area of the insulator and subjected to scanning electron microscopy (SEM) and energy dispersive X-ray spectroscopy (EDS) analysis on a Zeiss Supra 55 SEM (manufactured by Carl Zeiss Co., Ltd. in Oberkochen, Germany) equipped with an Oxford X-Max 20 EDS detector (manufactured by Oxford Instruments Co., Ltd. in Oxford, Britain) to determine the content and distribution of the pollutions on the surface. A Leica EMACE 200 fully automatic low-vacuum coating system was used to coat the sample with Pt to improve its surface conductivity.

A LIBS system assembled by our research group was used in the experiment. This LIBS system consists of a Nimma-900 laser system (wavelength: 1,064 nm, pulse width: 10ns, output frequency: 1 Hz, and output energy: 110 mJ), focal spot diameter of approximately 80 µm, laser energy density of 2.1883 × 10^11^ Watts/cm^2^, an Avantens spectrometer (available wavelength range: 200–650 nm) and a DG645 delay controller. The delay controller controls the interval between the output of the laser system and the acquisition of the spectrometer to effectively obtain an atomic emission spectrum evolved from a continuous background emission spectrum generated after plasma excitation under the action of the laser. The delay time was set to 3 µs in the experiment. The horizontal laser beam emitted by the laser system was reflected by a 45° mirror onto the vertical plane and focused by a convex lens onto the surface of the sample. The lens-to-sample distance was adjusted to position the sampling spot at the focal point of the convex lens. The spectral data acquired by the spectrometer were exported using the software Avasoft 8.8 (developed by Avantes Co., Ltd. in Apeldoorn, the Netherlands) and were subsequently processed.

In the experiment, kaolin clay was mixed with NaCl of different particle sizes at a 1:1 mass ratio. The shape of mixture is a circle with a diameter of 8 nm. Each mixture was compressed using a compression machine under compressive loads of 9t. and subsequently subjected to LIBS testing. Thus, the spectra of the Na in NaCl of different particle sizes were obtained. The NaCl particle size was determined using a laser particle size analyzer. In addition, the spectral intensity of NaCl obtained by LIBS was normalized to improve the analytical accuracy.

Spectrographic-grade NaCl samples (manufactured by Aladdin Industrial Co., Ltd. in Shanghai, China) were used in the experiment. Additionally, after sieving through <60, 60–100, 100–200 and 200–300 mesh stainless steel sieves, corresponding to particle sizes of >250, 150–250, 75–150 and 200–300 µm, respectively, NaCl samples of four different particle sizes were obtained (50 g of each type).

## 4. Conclusions

In this study, the microregional characteristics and element distributions of natural pollutions were analyzed with LIBS. The conclusions derived from this study are summarized as follows:

(1) Natural pollutions were obtained from the surface of an composite insulator from a 220kV transmission line. Through EDS, the main elements (Na, Mg, Si, Fe, O and C) composing the pollution sample were detected, which are common elements in natural pollutions. LIBS detected compositional elements of the pollution sample, meanwhile EDS failed to detect, thus, effectively reducing the element detection limit.
(2) A 110-mJ laser pulse was sufficient to penetrate the artificial pollutions on the surface of the insulator. With the accumulation of pulses, the relative spectral intensities of the common pollution elements on the LIBS spectrum gradually decreased.
(3) Artificial pollutions were prepared. The effects of the LIBS delay time and laser energy on the spectral signals were examined. The results showed that selecting a suitable delay time could improve the repeatability of data detection. In this study, the delay time was set to 3 µs. An increase in the laser energy increased the relative spectral intensity and RSD of each element. A suitable laser energy must be selected so that the laser does not harm the insulator substrate. In this study, the laser energy was set to 80 mJ, corresponding to a laser energy ablation density of 3.814 × 10^10^ Watts/cm^2^.
(4) The effects of the pollution properties (particle size and density) on the spectral signals were analyzed. A decrease in the particle size and an increase in the density of the sample both, improved the relative spectral intensities of the elements tested.

## Figures and Tables

**Table 1:** Energy dispersive X-ray spectroscopy (EDS) analysis results for the composition of natural pollutions on the surface of the insulator.

Element	C	O	Na	Mg	Al	Si	Ti	Fe
wt/%	13.63	35.8	0.34	0.31	6.03	17.95	21.78	4.17

**Table 2:** Wavelength of typical spectral lines for pollutions element by LIBS.

Element	C I	Mg II	Si I	Al I	Ti I
**Wavelength/nm**	247.856	279.553	288.158	309.271	359.871
**Element**	Ca II	Al I	Fe I	Na I	Na I
**Wavelength/nm**	393.366	396.152	425.079	588.995	589.592

**Table 3:** Test results obtained using the laser particle-size analyser

Particle Size/μm	NaCl				Kaolin Clay
	<60 Mesh	60–100 Mesh	100–200 Mesh	200–300 Mesh	
Distribution/50%	342.3	240.763	140.694	60.914	5.346
